# Population aging: opportunity for business expansion, an invitational paper presented at the Asia-Pacific Economic Cooperation (APEC) International Workshop on Adaptation to Population Aging Issues, July 17, 2017, Ha Noi, Viet Nam

**DOI:** 10.1186/s41043-018-0138-0

**Published:** 2018-04-10

**Authors:** Mary Beth Arensberg

**Affiliations:** Abbott Nutrition Division of Abbott, 3300 Stelzer Road, Columbus, OH 43219 USA

**Keywords:** Older adult population, Elderly consumers, Healthy aging, Malnutrition, Policies, APEC, Global

## Abstract

A longer life brings opportunities for older adults and their families as well as for their communities. Commercial businesses can be successful in innovating on these opportunities and achieving business expansion when they better understand the market dynamics and spectrum of older adults as consumers and view them more as assets rather than as burdens to society. While there is no “typical” older adult consumer, some traits, characteristics, and physical realities may be more common, including those related to family and community, the shopping experience, brand marketing and packaging, food and nutrition, and health. The opportunities of longer life are impacted by health and underscore the importance of positive, healthy aging-related behaviors like good nutrition and active lifestyles. Healthy aging also requires a sustained commitment and action from country leaders to formulate evidence-based polices--like systematic nutrition screening and intervention—and healthcare workforce training and education that can strengthen and support an active aging population. In addition, governments should consider engaging commercial businesses to help set sustainable policies that can advance products for older adults. Finally, governments should set national and local goals to incentivize commercial business development and investment in public-private partnerships to improve quality of care, promote healthy aging, and impact outcomes for noncommunicable diseases, ultimately benefitting population health for Asia-Pacific Economic Cooperation (APEC) countries.

## Introduction

While the world is rapidly aging, it is believed that in general, commercial businesses are unprepared to meet the needs of aging consumers. Specifically, in many markets around the world, there are gaps in the products and services that may be available compared to those that older consumers actually need and want. In a number of countries social services (most often provided by government and not-for-profit entities) may be available to help support older adults. However, developing specific products and services for the older adult market has not been a particularly strong focus of commercial businesses or the for-profit sector. In considering these opportunities, the aim of this paper is to review from a commercial business perspective the market dynamics in terms of the potential size, growth, and power of the older adult market; to describe unique characteristics of older adults as consumers that may be important for commercial businesses considering population aging as an opportunity for business expansion; and to outline areas for policy development and collaboration between the public and private sectors to support adaptation to population aging, particularly in nutrition and health.

This invited paper was prepared for and presented at the Asia-Pacific Economic Cooperation (APEC) International Workshop on Adaptation to Population Aging Issues, held July 17–18, 2017, in Ha Noi, Viet Nam. Because Abbott’s products include nutrition products, the APEC Workshop organizers asked that this paper include a nutritional perspective and that the paper be presented during a plenary business session on Population Aging and Economic Growth, with the objective that this paper offer a commercial outlook on the opportunities for business expansion that an aging population may provide. Thus, the target audience of the paper was APEC business executives and commercial leaders. APEC is an international forum for 21 Pacific Rim member economies that promotes free trade throughout the Asia-Pacific region. The 21 member economies are Australia; Brunei Darussalam; Canada; Chile; People’s Republic of China; Hong Kong, China; Indonesia; Japan; Republic of Korea; Malaysia; Mexico; New Zealand; Papua New Guinea; Peru; The Philippines; Russia; Singapore; Chinese Taipei; Thailand; The United States; and Viet Nam.

### Older adult market size, growth, and power

A commercial perspective on opportunities for business expansion starts with a review of market dynamics including the market size, growth, and power of the older adult market. This paper presents a brief overview since other presentations during the APEC Workshop included targeted details on aging and economic growth. By 2020, an estimated 13 countries will be “super-aged,” meaning more than 20% of their population will be aged 65 years or older. Japan already meets this definition today. By 2030, 21 more countries will join the ranks of the super-aged, including the APEC member economies of Canada, Hong Kong, New Zealand, Singapore, the Republic of Korea, and the U.S. Japan’s older adult population will continue to swell, reaching an estimated 30% aged 65 or older by 2030; Hong Kong will be close behind at 29% of their population aged 65 or older by 2030 [1].

The East Asia and Pacific region is said to have more older people today than anywhere else in the world and is aging more rapidly than any region in history [2]. China will move from an aging to an aged society in 25 years, Singapore and Thailand in 22 years and Viet Nam in only 19 years [3]. When considering the opportunity for business expansion in addressing an aging population, it is important to remember how the different regions across the world are at different “stages” of aging and which sectors are showing the most growth. For example in Japan and the Republic of Korea, 40% of their consumption growth is going to come from the upper end of the age spectrum, those aged 75 years and older. In contrast, in the U.S., it is projected that the most rapid consumption growth will come from those aged 60 to 75 years old [4].

Certainly, there is no doubt about the power of the older adult market, with older adults projected to spend $15 trillion annually by 2020 [5]. Further, those over aged 60 years will generate more than one third of global consumption growth [6] and more than half of all growth in urban consumption in the coming years [7]. In Northeast Asia, urban older adults are the *only* age group that is actually growing [7].

However, just as there are differences in how global regions are aging, there are also differences in how older adults in different regions may be spending. This is in part because growth is coming by the sheer size of the older adult segment, not necessarily by the wealth of the older adult segment. When looking at spending patterns, income is important and pension systems strongly influence this. In the U.S. for example, pension systems have shifted from a defined benefits to a defined contributions model, meaning there is a potential for wide variability in older adults’ pensions, depending on how much they chose to save over the course of their working careers.

There are also important differences in consumption patterns among older consumers. In Northeast Asia, traditionally the working-age group as well as those aged 60 years and older, have invested heavily in the younger generation. This is not expected to change and older adults in this region are often frugal in their spending patterns. In contrast, the Baby Boomers (those born from 1946 to 1964) in North America have typically saved less and consumed more than preceding generations in the region and this pattern will likely continue [7]. So even in comparing these two regions, there are central differences in how older adults may choose to spend their money.

### Older adults as consumers

To be effective in truly harnessing the growth of the older adult market, the APEC Workshop organizers identified that commercial businesses have a primary need to better understand older adults as consumers, Without this understanding, there are often gaps in the commercial products and services that may be offered compared to those that older consumers actually need and want. The definition of “older consumers” is complicated and dynamic, in part because 65 year olds are not 85 year olds and perhaps more importantly there is a wide spectrum even among a single age group in terms of income, health and social demographics, family structure and values, personal interests and experiences, and other factors. It is said that Baby Boomers will be the longest lived of any generation and may actually spend more time in retirement than they did working [4]. However, affluent graying Baby Boomers are also in denial about aging, with 61% feeling at least nine years younger than their actual age [4].

Those companies who will likely be most successful in meeting the needs and wants of older adult consumers will be those who are best able to understand the many facets and nuances of this population. While there is no “typical” older adult consumer, there are some traits, characteristics, and physical realities that may be more common among this age group compared to younger populations. Some of the more important considerations for commercial businesses include those related to family and community, the shopping experience, brand marketing and packaging, food and nutrition, and health. These are described in more detail below along with examples of commercial business innovations and opportunities for further development.

### Family and community

Having a family and a sense of community are central values for older adults. A U.S. Pew Research Center Social and Demographic Trends survey reported that when adults 65 years and older were asked what they valued most, 28% said spending more time with family and 25% said spending time with grandchildren [8]. When asked about their broader community, 83% of those aged 65 to 74 and 80% of those aged 75 and older said there are people other than family members who they rely on for social activities and companionship.

Such community relationships are important because the number and often percentage of older adults living alone is increasing in most countries. Even in societies where typically older parents live with their children, such as in Japan, traditional living arrangements are becoming less common [9]. Thus, although the number of surviving generations in a family has increased, these generations are more likely to live separately than they were in the past [10].

Women comprise the majority of the older population in virtually all countries, largely because globally women live longer than men. By 2025, both the proportion and number of older women are expected to soar from 107 to 373 million in Asia alone [11]. Many women are also less likely to have access to social protections such as pensions or health insurance plans which can then make life more challenging as they get older [12].

It is understandable then that older adults are very concerned about no longer being able to care for themselves, particularly in those lower and middle income countries where there may be more limited frameworks and resources to provide social supports. In a Nielsen survey of 30,000 internet respondents from 60 countries (including many APEC member economies), respondents were asked about their biggest fears: 58% reported the fear of not having self-reliance to care for basic needs, 57% reported the fear of losing physical agility, and 51% reported the fear of losing mental competence [13].

At the same time older adults are worried about maintaining their independence, they are also making significant shifts in how they use their time. Moving from a professional career to a more private life creates one of the greatest disruptions in life and means older adults often end up with lots of spare time that needs to be filled. For example, in the U.S. 90% of the growth in time spent in sports and outdoor activities will come from the aged 65 and older population [7]. A report on older Chinese consumers by Mintel found retirees had a “…strong propensity towards spending on experiences rather than possessions. Days out top the list of things extra money is spent on—with nearly half (44%) claiming to do this.” [14] How older adults pursue desirable and meaningful activities is said to depend largely on their wealth and their health [15].

Innovations commercial businesses may want to consider that can help meet the family and community needs of older adults include:

- Technologies that promote social connectivity and emotional health by supporting older adults’ interactions with their caregivers and broader communities, such as the development of IBM’s Internet of Caring Things (IoCT), which is a network of connected objects and cognitive systems that when applied to the aging “allow family members, doctors, and caregivers to proactively monitor the health and wellbeing of the world’s aging population.” [16] Another example is Stitch, a subscription service that connects older adults so they can socialize, travel, make friends, and find companions. With over 50,000 members in 50 cities worldwide, Stitch has the ultimate goals of improving the lives of older adults in every country in the world and providing an answer to social isolation and loneliness. And it has attracted interest from other businesses who want to partner, including travel and clothing companies [17].
- Brain training to help slow cognitive decline, like the promising results from the Advanced Cognitive Training for Independent and Vital Elderly (ACTIVE) study that examined how three brain training programs (focused on processing speed, memory and reasoning ability) impacted adults as they aged. At five years, those with training performed better on all three measures, at 10 years gains in reasoning ability and processing speed still persisted [18].
- Monetizing housing, including renovating and then renting out space in an older adult’s own home. In the U.S. adults aged 55 and older used to make up about one-third of the housing renovation market but now account for one-half of all home renovations [7]. In a 2015 study, Airbnb found people over age 60 were the fastest growing cohort of its hosts and worldwide about 260,000 of its two million listings were offered by those aged 60 or older [19]. Another renovation opportunity is home retrofitting to accommodate older adults continuing to live at home as they age. It is estimated to grow to potentially a $3.5 billion opportunity in the U.S. by 2020. And while there are over 650,000 remodelers in the U.S., only 5500 are “aging in place” certified, meaning they have special training to be experts in remodeling housing for older adults [20].

### The shopping experience

In developed economies, older adults spend relatively more than younger adults because they typically have higher incomes compared to other segments of the population. They are also more thoughtful in their shopping decisions, taking up to 25% more time in shopping for every dollar they spend [7]. In a report from ATKearney and The Consumer Goods Forum, it is explained that older adults devote more time to shopping in part because they have more available free time but also because they view shopping as a social experience. And they shop often, two-thirds of adults aged 70 to 80 report they shop twice or more a week, frequently on weekdays and early in the morning when stores may be less crowded. Proximity is an important reason older adults choose a specific store, particularly for the more aged consumers who also are more likely to prefer shopping in a smaller store [21].

Unfortunately, older consumers report that retail stores do not seem to be attuned to meeting their specific needs. In the Nielsen survey more than one third of global respondents commented it is difficult to find: electric shopping carts (41%), assistance with grocery bags (36%), and aisles dedicated to aging needs products (34%). Other comments included it is difficult to find benches to sit down on (29%) and difficult to find easy-to-reach shelving (23%) [13].

Older consumers also want quality and value. The ATKearney and Consumer Goods Forum report highlighted that older adults generally buy fewer items but spend more per item. The report commented as well that older adults “seek quality products, are loyal to brands, and are not particularly price-sensitive—even if their incomes are below average levels. This trend is even more pronounced with ageing”[21]. Brand loyalty may be due to many factors, including previous product experience and attachment, nostalgia, habit, and potentially aversion to change [22].

There is evidence too that the decision-making process may be different in older adults versus younger populations [23]. They may rely more on cognitive biases that help them make decisions quickly and efficiently [24]. In addition, they process information better at times of peak circadian arousal, which for older adults is typically in the morning, while younger adults reach peak circadian arousal in the afternoon or evening [25]. Further, older adults can be more vulnerable to exploitation and abuse. Kim and Geistfeld summarized older adult vulnerability as a combination of three factors: health status, cognitive ability, and social support, and identified in their research that the most vulnerable older adults are those who are more aged, less educated, ethnic minorities, and living in rural areas [26].

Based on the unmet needs older shoppers report, examples of retail innovation that may be helpful for commercial businesses include:

- Lawson, a convenience store in Japan that started to overhaul its stores in the mid-2000s, including widening aisles, lowering shelves, and emphasizing products and foods appealing to older adults. More recently they have added nursing-care consultation desks and also worked to ensure managers and advisors are available during all hours the store is open [27].
- CVS and Walgreens Drugstores in the U.S. made changes to help older adults navigate stores and read labels more easily. These changes included adjusting the height of shelves, adding carpeting, and attaching magnifying lenses to store shelves (to help customers read the fine print labels) [27].

### Brand Marketing and packaging

The ATKearney and Consumer Goods Forum report summed up their description of the older consumer by saying, “On the whole, mature consumers want and expect a sympathetic understanding of the realities of age, but they do not want to be treated as old or elderly” [21]. Probably no place is this more evident than in brand marketing and packaging.

Quite simply, older adults do not see themselves as “old.” The U.S. Pew trends survey reported 60% of those aged 65 years and older feel younger than their actual age. In older adults aged 65 to 74 years, one third reported feeling 10 to 19 years younger and 1 in 6 reported feeling 20 years younger. Further, when asked do they “feel old” most reported no; 78% of those aged 65 to 74 years old said no and 61% of those aged 75 years or older said no [8]. Yet at the same time, 51% of older adults report that ads do not reflect older consumers [13]. To deal with this dichotomy, marketing experts advise practicing “ageless marketing” by choosing models and situations that show vibrancy, with individuals active and engaged [28].

Contrary to popular opinion, technology use is common among the older population, with older adults outpacing younger cohorts when it comes to adopting new technology and online media [29]. The Nielsen survey reported more than one-third (37%) of global online respondents are already ordering groceries online for home delivery, and more than half (54%) were willing to try home delivery if it became available [13]. In the ATKearney and Consumer Goods Forum report, 69% of survey respondents had both fixed-line and mobile phones and while mobile phone usage declined sharply with age, half of respondents over age 80 said they used a mobile phone [21].

Older adults were also found to be heavy on-line users and shoppers; half of the ATKearney and Consumer Goods Forum report survey respondents used the Internet, with 20% taking advantage of it for shopping, research, and communication. The report also noted there is a strong divide between those who use the Internet and mobile technology and “those who will never get connected.” Right now that threshold is about age 72, but it goes up one year each year, as people stay connected. Thus, the report predicted the burgeoning older adult population could lead to an “e-commerce surge” in the future, and summarized “Based on historical adoption of digital technology and changing consumer behaviors, we tend to underestimate—and are only beginning to understand—the impact connected mature people will have on the future of the consumer industry and on society at large”[21].

Product packaging and labeling is another area where the needs of the older consumer may not be very well understood or met. Studies have shown that because of physical and social changes, “older people risk suffering embarrassment and anger, and even potential illness and serious injury as a result of difficulties with packaging” [30]. In the Nielsen survey, half of worldwide respondents said it was difficult to find product labels that were easy to read, 43% had trouble locating packages that were easy to open, 44% could not find smaller portion-sized food packing, and 43% had difficulty finding clearly labeled nutritional information on food packages. The survey also revealed important regional differences (Table 1) [13].

**Table 1:** Selected lowest/highest performing regions with products responding to aging needs, as rated by on-line Nielsen survey of older adults [13]

Lowest performing regions (% Older adults reporting products difficult to find)	Aging Need	Highest performing regions (% older adults reporting products easy to find)
Latin America (51%)	Easy-to-open product packages	Asia-Pacific & Middle East/Africa (54%)
Latin America (54%)	Clearly labeled nutritional information	North America (53%)
Asia-Pacific (50%)	Special nutritional diet foods	North America (52%)
Latin America (54%)	Smaller portion-sized packaging	Asia-Pacific (48%)

In short, older adults as consumers are often viewed as frail or dependent and thus judged by the faculties and functions they no longer have, instead of by the assets and capabilities they actually possess. But older adults are much more of an asset than a burden to society [31]. Changing this ageist attitude can help commercial businesses’ brands better target opportunities in the thriving older adult population.

Some of the innovations to support the development of new approaches in marketing, technology, and packaging that commercial businesses could learn from to help meet the needs of older adults include:

- The launch of the Modern Aging program by ACCESS Health International and NUS Enterprise to help create business in Singapore to serve the needs of elders and their caregivers. Modern Aging provides a four-month training program to entrepreneurs of all ages and backgrounds who wish to create new businesses to serve the needs of older adults [32]. “New products and services will become test-bedded in Singapore before being brought to the world” [33].
- Development of a virtual “aging suit” that simulates the effects of growing older, including metal hinges and bolts at joints to simulate stiffness and loss of mobility and a helmet that simulates impaired vision, hearing loss, and cognitive deterioration [34]. The suit could help marketers better understand the needs of older adults.
- Taking a different approach to beauty. Latin American women are the globe’s highest per income consumers of cosmetics and cosmetic surgery. It is predicted that aging Latin Americans will seek more natural and lasting beauty products and services, including exercise, dieting, and spa treatments to reverse or slow the effects of aging [35]. In Asia, portraying aging in a more positive light has been identified as an important marketing opportunity. Asian marketing initiatives for beauty products rarely feature older adults and anti-aging products are typically marketed to those aged 30 to 40 years old. One stand-out company who has found success is Shiseido, which has developed beauty products for those over age 50, has included older adults in its advertising, and has packaging with large font-size print [36].

### Food and nutrition

Not surprisingly, food and nutrition are important areas of focus for older adults. In fact in the Nielsen survey, eating healthy was one of respondents’ top four priorities in retirement, with 45% saying it was their most important priority. Other top priorities included staying physically and mentally fit (78%), spending time with family (58%) and maintaining an active social life (37%) [13]. In the U.S., five common trends have been identified in comparing the food and nutrition product interests of older adults to younger adults. Older adults are more likely to be: 1) health conscious, eat more fruits and vegetables and use nutrition supplements; 2) taste conscious, drawn to strong flavors; 3) package conscious, pay attention to packaging designs and ease of use; 4) value conscious, be concerned about nutritional value more than source of ingredients; and 5) convenience conscious, enjoy prepared foods, and eating out [37].

In addition to food product interests changing with age, there are changes in health and physiologic function that impact older adults’ nutrition needs too. A decrease in mental acuity, increase in disability, development and progression of chronic disease and other conditions, problems chewing and swallowing, depression and isolation, decreased appetite, sensory losses including sight, smell, taste, and other factors can all make it more difficult for older adults to shop, prepare, eat, and enjoy food. At the same time, nutrient needs are altered with aging, such as decreased energy needs and increased protein needs. From a policy perspective, there is an “increasing demand worldwide for [World Health Organization] WHO guidelines which competent national authorities can use to address the nutritional needs of their growing elderly populations”[11].

WHO has recognized that, “Under-nutrition, particularly loss of lean body mass, commonly underlies and exacerbates many conditions affecting older people, even in settings where food supplies are reliable and plentiful” [38]. The United Nations (UN) has targeted goals related to nutrition and older adults for well over 30 years, since the 1982 *Vienna International Plan of Action on Aging* [39]. More recently, the 2016 WHO report to UN Secretariat on *Multisectoral Action for a Life Course Approach to Healthy Aging: Draft Global Strategy and Plan of Action on Aging and Health*, stated “even in very advanced years, physical activity and good nutrition can have powerful benefits on health and well-being” [40]. Further, in the newest UN Sustainable Development Goals, aging and nutrition are relevant to many of the goals; older adult nutrition is specifically addressed in the target for Goal 2 (Target 2.2): “By 2030, end all forms of malnutrition…and address the nutritional needs of adolescent girls, pregnant and lactating women and older persons” [41]. However, to date UN agencies have developed very few--if any–-international nutrition programs specifically targeting older adults and their unique nutritional needs.

Ultimately, changing health and nutrition needs can put many older adults around the globe at risk for malnutrition. Yet, malnutrition is rarely adequately treated in hospitalized older adults [42]. Estimates show that 29 to 61% of older hospitalized patients may be malnourished [43]. Malnutrition can lead to increased disability and poor health outcomes. Globally, studies have repeatedly found direct relations between the degree of malnutrition and increased lengths of stay, treatment costs, and hospital re-admission rates [44]. A study investigating the burden-of-illness of disease-associated malnutrition in 15 diseases in China reported an annual economic burden of U.S. $66 billion (Chinese 447 billion). Investigators concluded, “This burden is sufficiently large to warrant immediate attention from public health officials and medical providers, especially given that low-cost and effective interventions are available” [45]. Similarly, a systematic literature review of disease-associated malnutrition in Latin American countries concluded: “Disease-related malnutrition is a highly prevalent condition that imposes a substantial health and economic burden on the countries of Latin America” [46].

Several innovative ways nutrition interventions are being tailored to meet the unique nutritional needs of older adults that could be bring opportunities for commercial businesses include:

- The research and development of specialized oral nutritional supplements with high levels of protein and specialized functional ingredients which have been shown to help older adults rebuild muscle for strength and energy [47].
- The expansion of meal services, including the regular delivery of ingredients and recipes for in-home preparation or the regular delivery of daily hot meals or frozen meals, including meals tailored to specific medical conditions [48].
- The innovation of 3D printed foods to create smooth and textured foods for older adults with difficulty chewing and swallowing. For example, work by researchers at the Commonwealth Scientific Industrial Research Organization in Brisbane, Australia is focusing on the role of 3D printed foods for dysphagia [49] and was recently featured at an Asia-Pacific 3D food printing conference in Melbourne, Australia [50].
- The research and development of electric cups and spoons which can create the impression of taste (saltiness, sourness, sweetness) and boost the flavor of bland foods. For example, engineers at the University of Singapore are developing a device to enhance the flavor of food and restore taste to those who may have lost it because of old age or disease treatment [51].

### Health

The health diversity seen in older adults is not random. A large part of the diversity comes from older adults’ physical and social environments and the impact of these environments on older adults’ opportunities and health behaviors [10]. Across APEC countries, the incidence of noncommunicable diseases (NCDs) including ischaemic heart disease, stroke, cancer, and diabetes has continued to increase as populations age. NCDs represent a significant economic and social burden; therefore, reducing the disability and complications that result from NCDs is central to helping limit healthcare costs. The Western Pacific region has been identified as the “epicenter” of the NCD epidemic, where the major NCDs—cardiovascular diseases, diabetes, cancers and chronic respiratory diseases—are responsible for 80% of all deaths in the region [52].

The UN 2030 Agenda for Sustainable Development recognizes NCDs as a major challenge for sustainable development [53] and WHO has developed a global monitoring framework to enable global tracking of progress in preventing and controlling NCDs and their risk factors [54]. NCDs are impacted in part by four modifiable risk factors--poor diet, tobacco use, insufficient physical activity, and harmful alcohol use--and thus programs are needed to help target reducing these risk factors.

Dementia is also a growing societal and health burden and the risk for dementia increases with age. Nearly 60% of the burden of dementia is concentrated in low- and middle-income countries and this is likely to escalate in coming years [55]. It was estimated over 48 million people worldwide were living with dementia in 2015, with that number expected to double every 20 years [56].

In 2012, WHO released a report to help governments, policymakers, and other stakeholders address the impact of dementia as an increasing threat to global health. The report noted “There is lack of awareness and understanding of dementia in most countries, resulting in stigmatization, barriers to diagnosis and care, and impacting caregivers, families and societies physically, psychologically and economically” [55]. Given that Alzheimer’s Disease is the most common type of dementia, that it is not impacted by available medications, and that about one-third of Alzheimer’s Disease may be attributed to five modifiable risk factors (Type 2 diabetes, smoking, physical inactivity, and overweight and obesity) [57] there is even further need for programs targeted to help reduce health risks and NCDs.

Healthcare innovations can help improve the sustainability of healthcare provision for older adults. Several innovations highlighted in a recent healthcare report by Marsh & McLennan Companies and the Asia Pacific Risk Center that may offer insights to commercial businesses include:

- Development of digital healthcare technologies, such as wearable health trackers that can potentially improve patient outcomes through increased treatment adherence and timely access to care.
- Application of Uber-style collaborative consumption business models for home nursing and caregiving to help decrease workforce shortages in long-term care.
- Development of robotic assistance to help improve the mobility of older adults, assist care workers and monitor older adults.
- Expanding applications and advancement in mobile phone technology, such as augmented reality to help increase engagement in health promotion [58].

The caregiving market is another area of tremendous opportunity for healthcare innovations and is expected to reach $72 billion by 2020 according to an AARP report. The report defines six main market segments and these may have application for commercial businesses development:

- Health and safety awareness: health vital alerts, diet and nutrition, medication management, personal safety monitoring, telehealth
- Care coordination: care planning, care professional engagement, records and benefits management, recovery support
- Daily essential activities: meals, home and personal care, home repair, delivery, transportation services
- Caregiver quality of life: respite and backup care, social support, health and wellness, financial and job security
- Social well-being: digital inclusion, life enrichment and empowerment, community networking, life companions
- Transition support: home retrofit services, long-term care insurance planning and provider referral, legal assistance, hospice and funeral planning [20].

### Areas for policy development and collaboration to meet older adult needs

Globally, life expectancy is increasing by about five hours a day, which translates into three additional months each year [59]. However, disability-free life expectancy is not increasing any faster than life expectancy, meaning there will continue to be a large and growing number of older adults living with chronic diseases and disabilities. Thus, systemic innovation is needed to mobilize community support, effectively use new technologies, and optimize environments to best support older adults [31]. Similarly, the APEC Workshop organizers identified that systemic innovation is important as governments build policy frameworks to support commercial business expansion and develop public-private partnerships focused on population aging.

### Policies in the health and nutrition sectors

Because the effects of aging are particularly significant for healthcare, there continues to be increased interest in sustainable policy reforms in the health and nutrition sectors, which could provide opportunities for commercial business expansion. One focus area is prevention. In their report on *Aging in East Asia and Pacific*, the World Bank Group identified “An overarching need is to transform health delivery systems by strengthening primary care services, shifting care away from acute care hospitals, reducing overprovision, and improving coordination among providers.” Further they identified “too little attention is paid to the prevention, early diagnosis, treatment, and control of health conditions” [2]. Similarly, a need for more attention to prevention is underscored in a WHO report on *The Health of Older People in Selected Countries of the Western Pacific Region*, where policy recommendations included:

- For developed economies, strengthen existing policies and public health interventions with the latest evidence to support lifestyle changes and risk factor prevention, improved health outcomes, decreased disease burden
- For emerging/transitioning and developing economies, implement public health efforts that support lifestyle changes and risk factor prevention [38].

As identified earlier in this paper, NCDs represent a primary area of importance for healthcare, and diet and nutrition are fundamental in the prevention and treatment of NCDs, particularly for older adults. The WHO Western Pacific Region report comments “Gains in population health may also be realized by increased focus of public health policy on assessing and meeting the immediate nutritional and food needs of older people. Prevention of malnutrition among older people has been shown to have positive health outcomes such as reduced susceptibility to disease and complications arising from co-morbid conditions and falls and reduced costs to health systems including reduced length of stay, and fewer re-admissions to care” [38].

A number of countries already include nutrition as a part of their aging-related policies. In 2015, HelpAge International commissioned a mapping of policy and legislation on aging and older people in 26 countries across the Asia and Pacific region. They reported that in 10 of 18 countries with national plans on aging, the nutrition needs of older people were included. Further, 15 of all 26 countries studied were found to have policies explicitly addressing older adults’ nutrition when mainstream documents were also considered. The various countries’ references to the importance of nutrition for healthy aging most often included providing education and information on nutrition [60].

While nutrition education and information are certainly important, preventing and treating malnutrition among older people requires a multi-sector approach. Recently, a multi-disciplinary collaboration in the U.S. released a *National Blueprint: Achieving Quality Malnutrition Care for Older Adults* that identified four primary goals and a number of strategies. The specific *Blueprint* goals are to:

- Improve quality of nutrition care practices for older adults
- Improve access to high quality nutrition care and services for older adults
- Generate clinical research on nutrition quality of care for older adults
- Advance public health efforts to improve nutrition quality of care for older adults [61].

The *Blueprint* can be used as an effective framework on a global scale to set national goals and incentives for addressing quality malnutrition care through developing policies that advance early screening, assessment, diagnosis, and intervention for older adults in hospitals and in the community. Commercial businesses could then consider for instance, expansion opportunities into home and community-based services by aligning with national goals and incentives to keep older adults healthy and well-nourished. One example is provided below.

### Public-private partnerships in nutrition

Tackling complex health problems such as malnutrition involves cross-sector alignment with government, nonprofit and philanthropic organizations, and corporate partners to actively coordinate actions, share lessons learned, and work toward the same goal with the same measures of success. Abbott is a global healthcare company, dedicated to helping people live their best lives through the power of health. The company has a history of successful partnerships in its core businesses of nutrition, diagnostics, and medical devices and works to help achieve common health goals through innovative solutions. Many of Abbott’s global partnerships with APEC member economies to address older adult malnutrition specifically align with the *Blueprint* goals, as listed in Table 2.

**Table 2:** Examples of Abbott global partnerships for malnutrition care of older adults that align with goals of the *National Blueprint: Achieving Quality Malnutrition Care for Older Adults* [61]

**Goal 1: Improve quality of nutrition care practices for older adults**
Canada: Canadian Malnutrition Task Force focused on education, data aggregation, development of best practices, and impacting policies related to malnutrition
U.S.: Malnutrition Quality Improvement Initiative focused on closing existing gaps in hospital-based malnutrition care through development/adoption of malnutrition electronic clinical quality measures and best practices; eQIP (Quality Improvement Program) web-based aggregate data system to collect nutrition care practice/hospital information and show areas of opportunity for quality improvement programs; Continuing education programs providing evidence-based education on malnutrition, nutrition interventions, and nutrition-focused physical assessment
Viet Nam: QIP (Quality Improvement Program) to help demonstrate value of proper malnutrition screening and interventions
**Goal 2: Improve access to high quality nutrition care and services for older adults**
Global: Total Nutritional Therapy hands-on clinical nutrition course for physicians and allied health professionals to help improve outcomes for geriatric patients
Viet Nam: Memoranda of Understanding partnership supporting the Ministry of Health’s effort to improve the nutritional status of Vietnamese people; Viet Nam Older Adult Health Check and Parent Companion Events offering free/comprehensive health checks and nutrition consultations to help support diet/lifestyle changes to enhance strength
**Goal 3: Generate clinical research on nutrition quality of care for older adults**
China: Research on the economic burden of disease-associated malnutrition; potential adverse impact of malnutrition on health/economic outcomes; malnutrition risk evaluation in hospitals
Taiwan: Research on the economic burden of disease-associated malnutrition; economic burden of malnutrition in end-stage renal disease patients on dialysis
U.S.: Nutrition Day study documenting prevalence of malnutrition in hospitals and impact on health outcomes; research on the economic burden of malnutrition at national/state levels and the economic burden of sarcopenia; study evaluating best in class protocols and effectiveness for quality improvement; survey of patient characteristics/healthcare utilization by community patients and health outcomes; establishment of interdisciplinary Center for Nutrition, Learning, and Memory in partnership with University of Illinois
**Goal 4: Advance public health efforts to improve nutrition quality of care for older adults**
U.S.: Defeat Malnutrition Today coalition that works to achieve recognition of malnutrition as key indicator and vital sign of older adult health risk and achieve greater focus on malnutrition through regulatory/legislative change; state malnutrition commissions to evaluate impact of malnutrition and evaluate/recommend state-level interventions; Community Malnutrition Resource Hub to provide web-based information, tools, and education resources on malnutrition for community based service providers

## Conclusion

A longer life brings opportunities for older adults and their families as well as for their communities. Commercial businesses can be successful in innovating on these opportunities when they better understand the market dynamics and the spectrum of older adults as consumers and view them more as assets rather than as burdens to society. The opportunities of longer life are impacted by health and a significant part of the diversity in older age is caused by the cumulative impact of health inequities across the life cycle. People are living longer but there is also increasing prevalence of NCDs.

Innovations in personalized medicine and older adults taking more active roles in healthcare decisions to live their fullest lives underscore the importance of positive health behaviors like good nutrition and active lifestyles. Improved nutrition can help preserve cognitive function, delay dependency and frailty, and thus underpin healthy aging. Healthy aging also requires a sustained commitment and action from country leaders to formulate evidence-based polices—like systematic nutrition screening and intervention—and healthcare workforce training and education that can strengthen and support an active aging population. Collective impact will be achieved through the commitment of key stakeholders from different sectors coming together to promote healthy aging. Governments should consider engaging commercial businesses to help set sustainable policies that can advance products for older adults. Finally, governments should set national and local goals to incentivize commercial business development and investment in public/private partnerships to improve quality of care and impact outcomes for NCDs, ultimately benefitting population health for APEC countries.

## Abbreviations

ACTIVE: Advanced Cognitive Training for Independent and Vital Elderly
APEC: Asia-Pacific Economic Cooperation
IoCT: Internet of Caring Things
NCD: Noncommunicable Disease
UN: United Nations
WHO: World Health Organization
